# Macitentan inhibits the transforming growth factor-β profibrotic action, blocking the signaling mediated by the ETR/TβRI complex in systemic sclerosis dermal fibroblasts

**DOI:** 10.1186/s13075-015-0754-7

**Published:** 2015-09-10

**Authors:** Paola Cipriani, Paola Di Benedetto, Piero Ruscitti, Daniela Verzella, Mariafausta Fischietti, Francesca Zazzeroni, Vasiliki Liakouli, Francesco Carubbi, Onorina Berardicurti, Edoardo Alesse, Roberto Giacomelli

**Affiliations:** Department of Applied Clinical Sciences and Biotechnology, Rheumatology Unit, School of Medicine, University of L’Aquila, Delta 6 Building, Via dell’Ospedale, 67100 L’Aquila, Italy; Department of Applied Clinical Sciences and Biotechnology, University of L’Aquila, Coppito 2, 67100 L’Aquila, Italy

## Abstract

**Introduction:**

Systemic sclerosis (SSc) is a complex and not fully understood autoimmune disease associated with fibrosis of multiple organs. The main effector cells, the myofibroblasts, are collagen-producing cells derived from the activation of resting fibroblasts. This process is regulated by a complex repertoire of profibrotic cytokines, and among them transforming growth factor beta (TGF-β) and endothelin-1 (ET-1) play a major role. In this paper we show that TGF-β and ET-1 receptors co-operate in myofibroblast activation, and macitentan, an ET-1 receptor antagonist binding ET-1 receptors, might interfere with both TGF-β and ET-1 pathways, preventing myofibroblast differentiation.

**Methods:**

Fibroblasts isolated from healthy controls and SSc patients were treated with TGF-β and ET-1 and successively analyzed for alpha smooth muscle actin (α-SMA) and collagen (Col1A1) expression and for the Sma and Mad Related (SMAD) phosphorylation. We further tested the ability of macitentan to interfere with these process. Furthermore, we silenced ET-1 and endothelin-1 receptor A expression and evaluated the formation of an ET-1/TGF-β receptor complex by immunoprecitation assay.

**Results:**

We showed myofibroblast activation in SSc fibroblasts assessing the expression of α-SMA and Col1A1, after stimulation with TGF-β and ET-1. Macitentan interfered with both ET-1- and TGF-β-induced fibroblast activation. To explain this unexpected inhibitory effect of macitentan on TGF-β activity, we silenced ET-1 expression on SSc fibroblasts and co-immunoprecipitated these two receptors, showing the formation of an ET-1/TGF-β receptor complex.

**Conclusions:**

During SSc, ET-1 produced by activated endothelia contributes to myofibroblast activation using TGF-β machinery via an ET-1/TGF-β receptor complex. Macitentan interferes with the profibrotic action of TGF-β, blocking the ET-1 receptor portion of the ET-1/TGF-β receptor complex.

## Introduction

Systemic sclerosis (SSc) is characterized as an immune dysregulation and vascular injury that generally precedes and contributes to the development of fibrosis. During the disease, the tightly regulated and self-limited response to injury that normally leads to tissue regeneration is subverted into fibrosis, with disruption of tissue architecture and loss of functional integrity. Once started, fibroblast (FB) activation, leading to collagen and alpha smooth muscle actin (α-SMA) upregulation, is amplified through multiple feedforward loops, generated as a consequence of tissue damage, as well as hypoxia and oxidative stress [1]. Although the pathophysiological mechanisms of SSc fibrosis is still largely unknown, injured endothelial cells (ECs) and pericytes may play an pivotal role in this process due to their ability to transdifferentiate toward activated myofibroblasts [2–4], producing increased amounts of collagen [5–7]. In this setting, both endothelin-1 (ET-1) and transforming growth factor beta (TGF-β) have been shown to play a key role in this process. ET-1, one of the three members of ET family, is known to have a strong vasoconstrictive activity and is further involved in vascular remodeling. The molecule is largely released from ECs [8, 9] and binds two different receptors, endothelin-1 receptor A and B (ETAR and ETBR) [10]. ET-1 is involved in SSc vasculopathy, including digital ulcers and pulmonary arterial hypertension (PAH) [11, 12], and its targeting represents an important therapy for these vascular manifestations. Recently, ET-1 has been implicated in the fibrotic process in different organs, such as skin, lung and heart [13, 14], although the detailed mechanism of the ET-1 effects still needs to be clarified.

TGF-β is the main profibrotic cytokine involved in the pathogenesis of fibrosis in SSc, inducing FB activation, collagen production and subsequent remodeling of the extracellular matrix (ECM) [15]. TGF-β, after binding with specific receptors, induces the formation of a type II–type I receptor complex, in which the constitutively active type II receptor (TβRII) phosphorylates and activates the type I receptor (TβRI). Successively, the signal is transduced to the nucleus by members of the Sma and Mad Related (SMAD) family. In the past years, the induction of SMAD1/5 phosphorylation by TGF-β was considered to be specific to ECs [16, 17], although it has been recently shown that TGF-β may induce phosphorylation of both SMAD1 and SMAD5, together with phosphorylation of SMAD2/3, in different cell lines, such as epithelial cells, FBs and cancer-derived cell lines [18].

It has been recently suggested that TGF-β, although highly activated, may be not sufficient to support the persistent fibrotic responses noted in SSc patients [19], but that it is working in synergy with other extracellular ligands, such as connective tissue growth factor and ET-1 [19]. Over the past years, different groups [15, 20] have pointed out roles for TGF-β and ET-1 on the FB acquisition of phenotype and function associated with myofibroblasts.

In this work, we provide evidence that macitentan (MAC), a novel specific ETAR/ETBR antagonist [21], prevents SSc FB activation induced by TGF-β, blocking protein collagen 1 alpha 1 (Col1A1) production and α-SMA expression. We further show that its inhibitory effect is due to the presence on the FB surface of a functional endothelin receptor (ETR)/TβRI complex, whose ETR portion is recognized and blocked by MAC.

Understanding the mechanism regulating ET-1/TGF-β receptor interaction, may provide new future possibilities in the treatment of fibrosis, a situation still needing effective therapies.

## Methods

### Patients and FB isolation

After approval from the San Salvatore University Hospital ethics committee and written informed consent from patients, FBs were obtained from 10 SSc patients with the diffuse form of disease of recent onset (disease duration less than 3 years calculated since the first nonRaynaud’s symptom of SSc) [22, 23] by skin biopsies. Demographic and clinical characteristics of the patients are shown in Table 1.

**Table 1:** Clinical and demographic features of the 10 diffuse systemic sclerosis patients

Sex/age (years)	Year of SSc onset/disease duration at skin biopsy (years)	MRSS/score at skin biopsy	Autoantibodies	Lung involvement HRCT/PFT	Heart involvement/Scleroderma renal crisis	Raynaud’s phenomenon/digital ulcers
F/46	2010/2	12/2	ANA/Scl-70	Normal/Normal	Normal/No	Yes/No
F/21	2009/3	13/1	ANA/Scl-70	Normal/Normal	Normal/No	Yes/Yes
F/31	2011/1	13/2	ANA/Scl-70	Normal/Normal	Normal/No	Yes/Yes
F/36	2010/2	11/2	ANA/Scl-70	Normal/Normal	PAH/No	Yes/Yes
M/20	2010/2	11/1	ANA/Scl-70	Normal/Normal	Normal/No	Yes/No
F/41	2010/2	15/2	ANA/Scl-70	Normal/Normal	Normal/No	No/No
F/30	2010/2	10/1	ANA/Scl-70	Normal/Normal	Normal/No	Yes/No
F/21	2010/2	09/1	ANA/Scl-70	Normal/Normal	Normal/No	Yes/No
F/31	2009/3	14/1	ANA/Scl-70	Normal/Normal	Normal/No	Yes/No
F/42	2009/3	16/2	ANA/Scl-70	Fibrosis/Normal	Normal/No	Yes/No

The internal organ involvement refers to the time of biopsy. *ANA* antinuclear antibodies, *F* female, *HRCT* high-resolution computed tomography, *M* male, *MRSS* modified Rodnan skin thickness score (maximum possible score 51), *PAH* pulmonary arterial hypertension, *PFT* pulmonary function test, *Scl-70* anti-topoisomerase, *SSc* systemic sclerosis

Patients discontinued corticosteroids, oral vasodilators, intravenous prostanoids or other potentially disease-modifying drugs at least 1 month before biopsies. None received immunosuppressants.

Frozen FB samples obtained from age-matched healthy women donors of skin samples, for research purposes, were used as controls.

Biopsy samples (1 × 0.5 cm) of the involved forearm skin (skin score 1/2 at the biopsy site) were washed with phosphate-buffered saline (Life Technologies, CA, USA) and four explants were placed into a 50 ml tube containing 15 ml collagenase (Sigma-Aldrich, MO, USA) and then digested for 2 h at 37 °C. Cells were cultured in Dulbecco’s modified Eagle’s medium (DMEM; GIBCO, CA, USA), supplemented with 10 % fetal bovine serum (Standard South America origin, Lonza, MD, USA), 2 mmol/l L-glutamine (EuroClone, Milan, Italy), and 100 U Penicillin and 1,000 U Streptomycin (Biochrom AG, FL, USA). At 80 % confluence the FBs were split and subcultured. Third-passage (P3) FBs were analyzed for the surface expression of S100A4.

### FBs treatment with TGF-β, ET-1 and MAC

To establish the optimal concentration of TGF-β (R&D, USA), ET-1 (Sigma-Aldrich) and MAC in our system, a dose–response curve was performed on α-SMA expression (data not shown), using P3 FBs obtained from both one control and one patient.

Each experiment was performed in triplicate, and the optimal stimulation dose for TGF-β was assessed to be 10 ng/ml, for ET-1 it was assessed to be 200 nM, and for MAC, 1 μM.

To assess α-SMA and Col1A1 expression, FBs were treated under the following conditions: 1) untreated (UT) FBs; 2) FBs + TGF-β (10 ng/ml); 3) FBs pretreated (1 h) with MAC (1 μM), before being treated with TGF-β; 4) FBs + ET-1 (200nM); 5) FBs pretreated (1 h) with MAC (1 μM), before being treated with ET-1. The experimental conditions were applied for 6 days in DMEM with 1 % fetal bovine serum and the medium was changed every 2 days.

To assess SMAD1/5, SMAD2/3 phosphorylation and immunoprecipitation experiments, FBs were treated under the following conditions: 1) UT FBs; 2) FBs + TGF-β (10 ng/ml); 3) FBs pretreated (1 h) with MAC (1 μM), before being treated with TGF-β; 4) FBs + ET-1 (200nM); 5) FBs pretreated (1 h) with MAC (1 μM), before being treated with ET-1. The experimental conditions were applied for 24 h in DMEM with 1 % fetal bovine serum.

### Western blot

In order to perform Western blot assays, FBs cells were pelleted, washed twice with phosphate-buffered saline, lysed in lysis buffer (Tris Cl pH 7.4 10 mM, NaCl 100 mM, EDTA 1 mM, EGTA 1 mM, Triton X 1 %, NaF 5 mM, Na_3_VO_4_ 1 mM, PMSF 1 mM, leupeptin 10 μg/mL, aprotinin 10 μg/mL, Roche tablet inhibitor) and the protein concentration was calculated by Bradford protein assay reagent (Bio-Rad, USA). Proteins (50 μg) were separated by SDS-PAGE and transferred to nitrocellulose membranes. After 1 h at room temperature in blocking buffer (5 % nonfat milk in Tris-buffered saline/1 % Tween 20 (TBS/T)) the membranes were washed three times for 5 min each in TBS/T, and incubated overnight at 4 °C with the primary antibodies: α-SMA, ET-1 (Abcam, USA), Col1A (Santa Cruz Biotechnology, USA), Phospho-SMAD1/5, Phospho-SMAD2/3 (Cell Signaling, USA) diluted in 5 % bovine serum albumin in TBS/T. Following three washes with TBS/T, horseradish peroxidase-conjugated secondary antibodies (Santa Cruz Biotechnology) diluted in blocking buffer were added for 30 min at room temperature and washed three times with TBS/T. The detection was performed by enhanced chemiluminescence detection ECL reaction (Amersham Pharmacia Biotechnology, USA). All the results were normalized to the levels of proteins of UT healthy control (HC) FBs and normalized to the actin signal (Sigma-Aldrich). Immunoreactive bands were quantified with densitometry using ImageJ software (NIH, Bethesda, USA).

### siRNA assay

In order to silence both ET-1 and ETAR expression, SSc FBs were transfected with Silencer Select ET-1-siRNA and ETAR-siRNA (Life Technologies) or with Silencer Select Negative Control non-targeting siRNA (scr) (Life Technologies) using Lipofectamine™ 3000 (Life Technologies, USA).

Transfection was performed according to the manufacturer’s instructions. Briefly, FBs were plated at 1 × 10^4^ cells per cm^2^, 24 h prior to transfection. Cultures were incubated for 24 h with 25 pmol siRNA in 2 mL OptiMem. After incubation, plates were washed and cells were allowed to recover in normal growth conditions (10 % DMEM) for 24 h post-transfection.

### Immunoprecipitation

Cells were washed three times with cold phosphate-buffered saline and solubilized in 200 μl lysis RIPA buffer (SDS 0.1 %, NP-40 1 %, Na_3_VO_4_ 0.5 %, PMSF 1 mM, aprotinin 10 μg/ml, Roche tablet inhibitor). After centrifugation at 12,000 rpm for 10 min, 0.5 mg protein was subjected to immunoprecipitation. Specific anti-TβRI antibody (Santa Cruz Biotechnology) was added and rocked at 4 °C for 1 h; 30 μL protein A/G beads (Santa Cruz Biotechnology) was added and the sample was rocked over night at 4 °C. For Western blotting, anti-Phosphoserine (BD Biosciences, CA, USA) and anti-ETAR (Santa Cruz Biotechnology, USA) were used.

### Statistical analysis

GraphPad Prism 5.0 software was used for statistical analyses. Results are expressed as median (range). Due to the nonparametric distribution of our data, the Mann–Whitney U test was used as appropriate for analyses. Statistical significance was expressed by a *p* value ≤0.05.

## Results

### MAC inhibited the Col1A1 and α-SMA upregulation induced by both TGF-β and ET-1

**Fig. 1:**
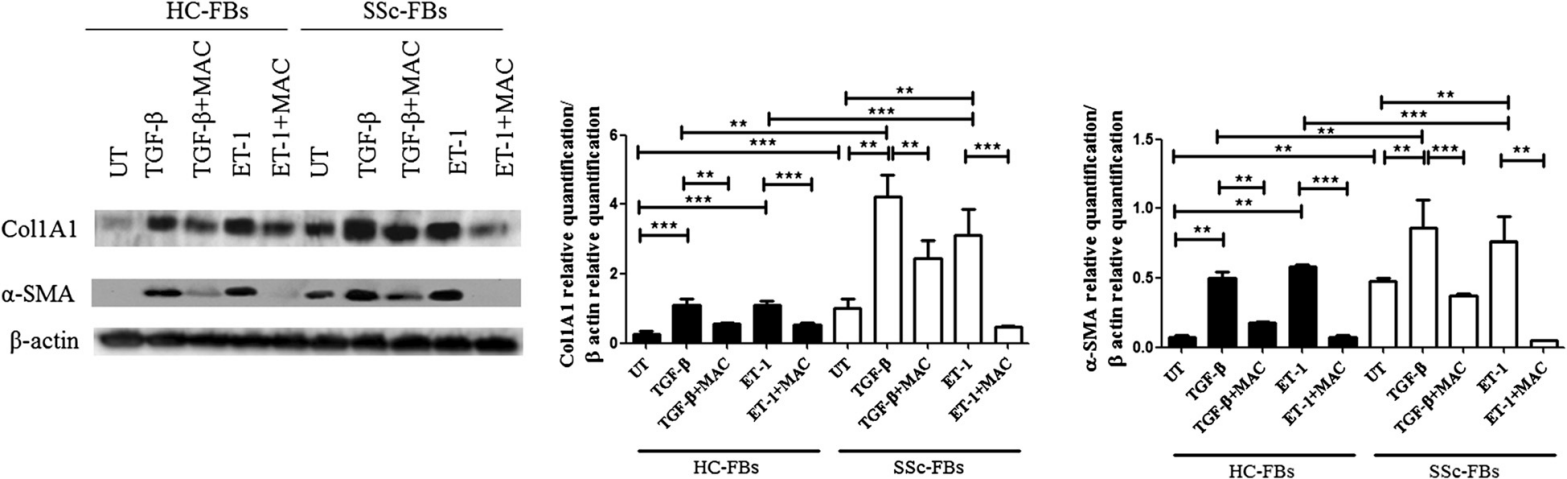
Macitentan (MAC) inhibited both transforming growth factor beta (TGF-β) and endothelin-1 (ET-1) effects. Protein and gene expression of collagen 1 alpha 1 (Col1A1) and alpha smooth muscle actin (α-SMA) in fibroblasts (FBs). Under untreated (UT) conditions the basal expression of Col1A1 and α-SMA proteins was significantly higher in systemic sclerosis (SSc) FBs when compared to healthy control (HC) FBs. The TGF-β and ET-1 treatment induced a significant upregulation of both Col1A1 and α-SMA expression when compared to UT FBs and this increase was significantly higher in SSc FBs. MAC significantly blocked both TGF-β and ET-1 effects. Pictures are representative of all experiments. Protein bands were quantified by densitometry and the values are expressed as protein relative quantification/β actin relative quantification. **p = 0.0002, ***p = 0.0001

Figure 1 shows that under UT conditions the basal expression of Col1A1 and α-SMA protein was significantly higher in SSc FBs when compared to HC FBs. The TGF-β and ET-1 treatment induced a significant upregulation of both Col1A1 and α-SMA expression when compared to UT FBs, and this increase was significantly higher in SSc FBs. MAC significantly blocked both TGF-β and ET-1 effects. The densitometry analysis of Western blot is reported in Fig. 1.

### TβRI activation in SSc FBs is blocked by MAC

**Fig. 2:**
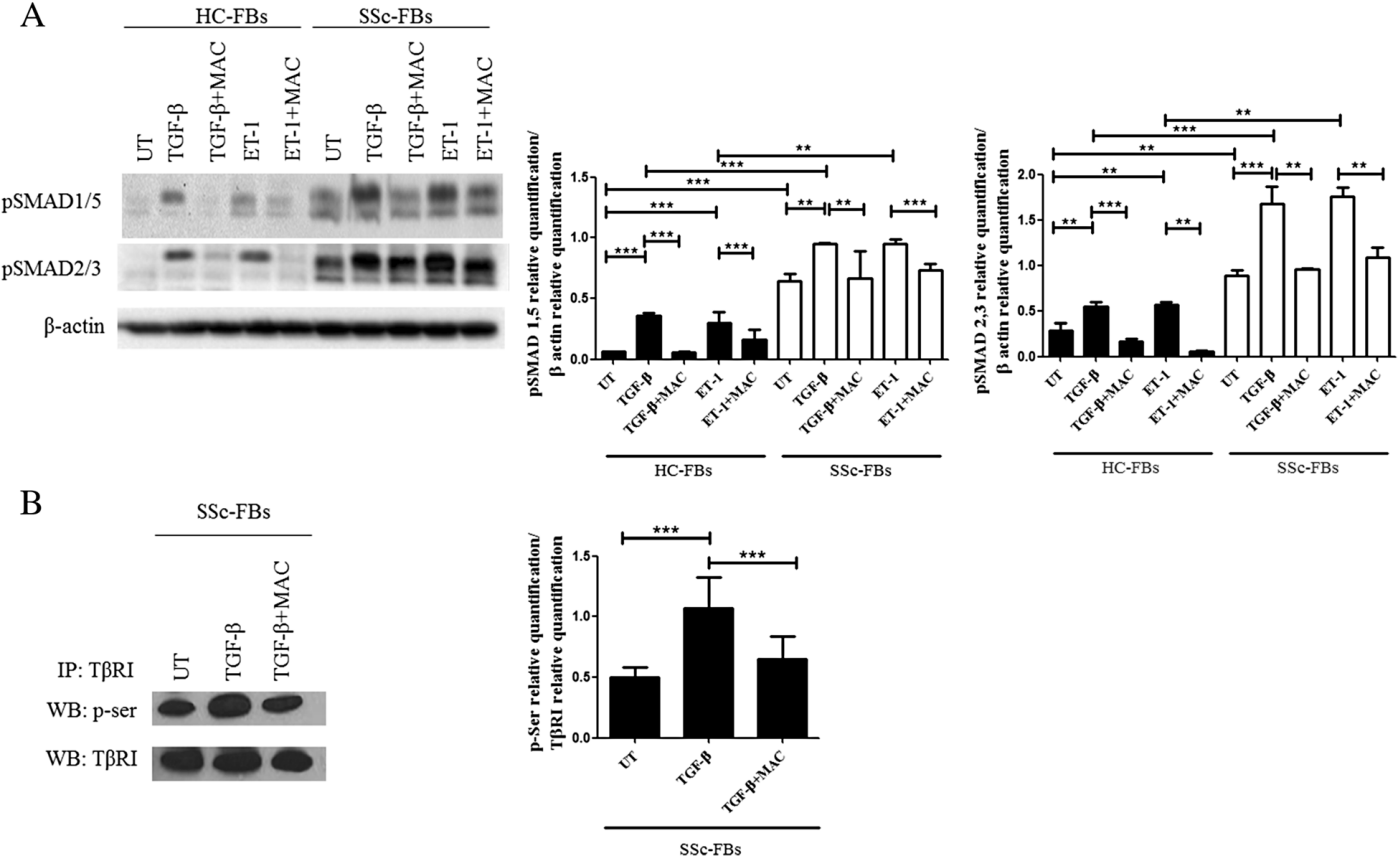
Macitentan (MAC) blocked transforming growth factor beta type I receptor (TβRI) activation. **a** Protein expression of phospho-Sma and Mad Related (pSMAD)1/5 and pSMAD2/3. Transforming growth factor beta (TGF-β) and endothelin-1 (ET-1) effects induced a significant increase of both SMAD2/3 and SMAD1/5 phosphorylation. MAC significantly blocked both TGF-β and ET-1 effects. The phospho-SMAD levels were significantly higher in systemic sclerosis (SSc) patients. Pictures are representative of all experiments. Protein bands were quantified by densitometry and the values are expressed as protein relative quantification/β actin relative quantification. **b** TβRI was immunoprecipitated (IP) and its phosphorylation was assessed by Western blot (WB). The immunoprecipitation assay showed a serine phosphorylation in TβRI after TGF-β treatment. MAC significantly inhibited the TβRI phosphorylation. Immunoprecipitated protein bands were quantified by densitometry and the values are expressed as protein relative quantification/TβRI relative quantification. **p = 0.0002, ***p = 0.0001. *UT* untreated

Figure 2a shows that UT SSc FBs displayed an increased SMAD1/5 and SMAD2/3 phosphorylation when compared to UT HC FBs. TGF-β and ET-1 induced a significant increase of both SMAD1/5 and SMAD2/3 phosphorylation in SSc and HC FBs. The level of phosphorylation was higher in SSc FBs. Although MAC is an ET-1 antagonist, the Western blot showed that the drug significantly inhibited both ET-1 and TGF-β effects. Furthermore, we evaluated the phosphorylation of TβRI on SSc FBs before and after TGF-β treatment, and our results show that MAC modulates the phosphorylation level of TβRI, induced by TGF-β treatment (Fig. 2b).

### SMAD phosphorylation is inhibited by MAC in SSc FBs transfected with ET-1 siRNA

**Fig. 3:**
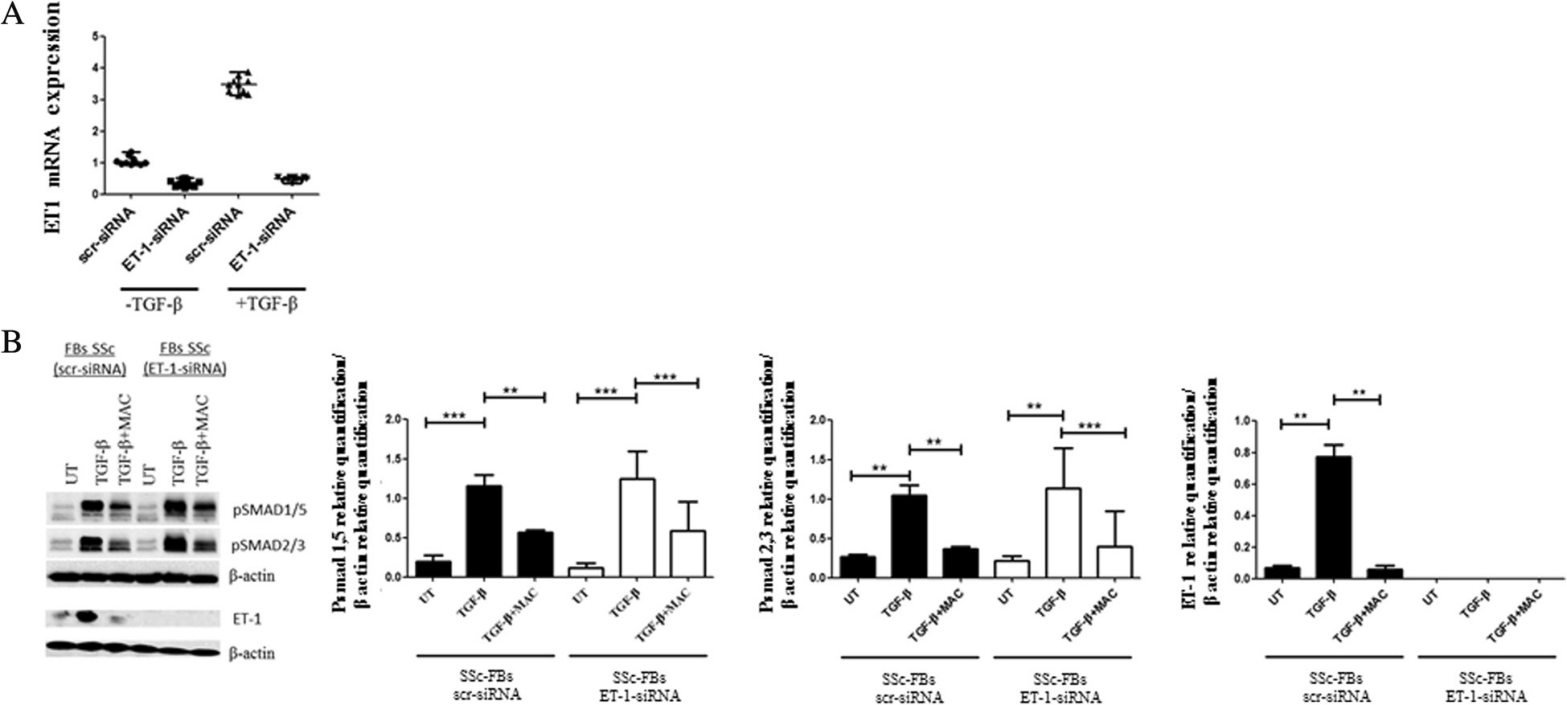
Macitentan (MAC) effects in systemic sclerosis (SSc) fibroblasts (FBs) transfected with endothelin-1 (ET-1) siRNA. **a** SSc FBs were transfected with specific ET-1 siRNA or nontargeting scramble scr-siRNA, and ET-1 expression was evaluated by quantitative RT-PCR. The cells transfected with ET-1 siRNA showed a decreased expression of ET-1 gene, when compared with cells transfected with scr-siRNA. The transforming growth factor beta (TGF-β) stimulus induces a significant increase in ET-1 expression in SSc FBs treated with scr-siRNA, but in SSc FBs treated with ET-1 siRNA; TGF-β was unable to induce an ET-1 increase. **b** Western blot of phospho-Sma and Mad Related (pSMAD)1/5, pSMAD2/3 and ET-1 proteins. In SSc FBs treated with scr-siRNA, TGF-β induced a significant increase in SMAD phosphorylation and MAC inhibited this effect. Mirroring these data, MAC inhibited the TGF-β effect in SSc FBs treated with ET-1 siRNA. In SSc FBs transfected with ET-1 siRNA, ET-1 protein was not expressed. Pictures are representative of all experiments. Protein bands were quantified by densitometry and the values are expressed as protein relative quantification/β actin relative quantification. **p = 0.0002, ***p = 0.0001. *UT* untreated

To inactivate ET-1 gene product in SSc FBs at a molecular level, we transfected SSc FBs with ET-1 siRNA or scrambled control siRNA (scr-siRNA). ET-1 siRNA efficiently (>70 %) knocked down ET-1. Figure 3a shows that TGF-β stimulus induces a significant increase in ET-1 expression in SSc FBs treated with scr-siRNA, but in SSc FBs treated with ET-1 siRNA, TGF-β was unable to induce an ET-1 increase. The Western blot analysis confirmed the mRNA results (Fig. 3b).

In SSc FBs treated with scr-siRNA, TGF-β induced a significant increase in SMAD phosphorylation, and MAC inhibited this effect. Mirroring these data, MAC inhibited the TGF-β effect also in SSc FBs treated with ET-1 siRNA (Fig. 3b).

### ETAR co-immunoprecipitates with TβRI

**Fig. 4:**
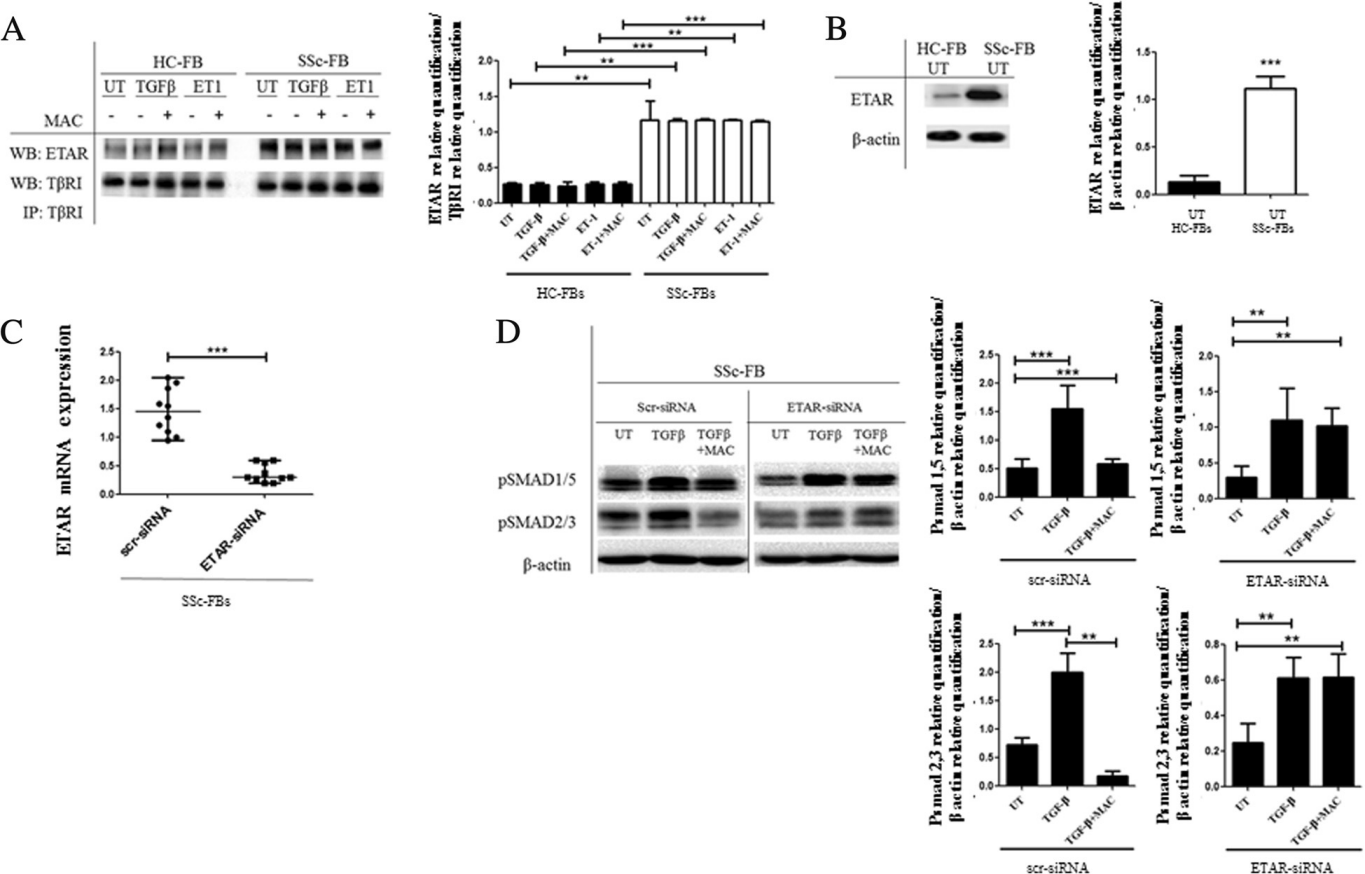
Transforming growth factor beta type I receptor (TβRI)/endothelin-1 receptor A (ETAR) co-immunoprecipitation. **a** TβRI was immunoprecipitated (IP) and its association with ETAR was assessed by Western blot (WB). The immunoprecipitation assay showed an association between ETAR and TβRI, independent of both transforming growth factor beta (TGF-β) and endothelin-1 (ET-1) stimulation. In systemic sclerosis (SSc) fibroblasts (FBs), the levels of co-immunoprecipitated receptors were significantly higher when compared with healthy control (HC) FBs. Blot was representative of all the experiments. Co-immunoprecipitated protein bands were quantified by densitometry and the values are expressed as protein relative quantification/TβRI relative quantification. **b** Western blot of ETAR protein. In SSc FBs, ETAR expression was significantly higher when compared with HC FBs. Blot was representative of all the experiments. The protein bands were quantified by densitometry and the values are expressed as protein relative quantification/β actin relative quantification. **c** SSc FBs were transfected with specific ETAR siRNA or nontargeting scramble scr-siRNA, and ETAR expression was evaluated by quantitative RT-PCR. The cells transfected with ETAR siRNA showed a decreased expression of ETAR gene, when compared with cells transfected with scr-siRNA. **d** Western blot of phospho-Sma and Mad Related (pSMAD)1/5 and pSMAD2/3. In SSc FBs treated with scr-siRNA, TGF-β induced a significant increase in SMAD phosphorylation, and macitentan (MAC) inhibited this effect. In SSc FBs treated with ETAR siRNA, TGF-β induced a significant increase in SMAD phosphorylation, and MAC failed to inhibit this effect. Pictures are representative of all experiments. Protein bands were quantified by densitometry and the values were expressed as protein relative quantification/β actin relative quantification. **p = 0.0002, ***p = 0.0001. *UT* untreated

Although ETAR has been shown to heterodimerize with other members of the ETR subfamily, heterodimerization with TβRI has not yet been described. Surprisingly, we found that the two receptors can be co-immunoprecipitated from FBs in the absence of ligand. This association was detected by immunoprecipitating the TβRI and staining Western blots with ETAR (Fig. 4a). No further increase in the amount of co-immunoprecipitating receptor with the addition of other ligands, such as TGF-β or ET-1, was observed. Of note, in SSc FBs, the ETAR levels that co-immunoprecipitated were significantly higher, when compared to HC FBs. These data parallel the evidence that ETAR expression in SSc FBs was significantly higher than ETAR expression in HC FBs (Fig. 4b).

### SMAD phosphorylation is not blocked by MAC in SSc FBs transfected with ETAR siRNA

To clarify the exact function of ETRs on the ETAR/TβRI complex, we transfected the SSc FBs with ETRA siRNA or scr-siRNA. In Fig. 4c, we show that, in ETAR-siRNA-treated SSc FBs, a transient silencing of ETAR was observed when compared to cells treated by negative scr-siRNA (silencing >70 %). In SSc FBs treated with ETAR siRNA, pSMADs were significantly increased after TGF-β treatment; of note, MAC lacks the ability to inhibit SMAD phosphorylation, such as reported in SSc FBs transfected with scr-siRNA (Fig. 4d).

## Discussion

Fibrosis of skin and internal organs is the dominant feature of SSc and, to date, no therapy has been shown to revert or arrest the progression of fibrosis. Myofibroblasts are generally considered to be the major effector cells responsible for the fibrotic events in the disease, since they contribute to the increased synthesis and contraction of the ECM, which is typical of this disorder. In fact, during SSc, FBs may switch from a form that displays a quiescent phenotype toward a proliferating, matrix-producing and contractile phenotype. Although the origin of myofibroblasts is still a matter of debate, recent studies indicate that bone marrow-derived mesenchymal progenitors undergo myofibroblast differentiation in damaged tissues [24] and we recently demonstrated that perivascular cells might contribute to myofibroblast generation under pathologic stimuli [5–7].

In the fibrotic process, TGF-β seems to play a pre-eminent role [25–27]. This molecule exerts its action through the induction (or repression) of many genes that function as downstream targets and contribute to its effects in a coordinated way [28]. Its action is mediated by SMAD family molecule phosphorylation and inhibiting these downstream products blocks TGF-β effects [18]. It has been reported that, in animal model of skin fibrosis, TGF-β was able to induce transcriptional activation of the ET-1 gene in dermal FBs [15], suggesting that these cells possess an active TGF-β/ET-1 axis and both these molecules may modulate fibrogenic responses. Furthermore, their synergistic effect seems to be particularly complex and a co-operation between their own receptors has been hypothesized.

Our results showed that, in skin FBs, ET-1, mirroring the TGF-β behavior, was able to induce myofibroblast activation, as shown by the increased expression of ColA1 and α-SMA. Several reports [20, 29–31] showed that ET-1 induces a profibrotic phenotype in FBs via the increase of ECM protein expression and the decrease of matrix metalloproteinase expression, suggesting that blocking ET-1 signaling by bosentan, a dual ET-1 receptor antagonist, could be a potential therapeutic strategy for fibrotic disorders [29]. The main studies on the anti-fibrotic effect of ETR antagonists on SSc FBs were reported by Shi-Wen et al. [32–35], showing that bosentan may suppress the expression of α-SMA, type I collagen, fibronectin, and CCN2 in SSc lung FBs and that ET-1 acts as a downstream mediator of TGF-β in human lung FBs. Lagares et al. [15] repeated these results in dermal FBs both in in-vitro experiments and in in-vivo animal models. Recently, the ability of MAC to prevent the ET-1 profibrotic effects on SSc FBs has been demonstrated [20]. Thus, ET-1 seems to be strongly involved in the fibrotic process, and blocking its activity by specific receptor antagonists may be helpful in the treatment of fibrotic disorders, although the detailed molecular mechanism of action of these new drugs still needs to be clarified.

In the present work, we showed that ET-1 performs its action sharing the TGF-β signaling pathway. In fact ET-1 stimulation led to SMAD phosphorylation and MAC blocking the ET-1-induced SMAD signaling pathway, preventing the induction of myofibroblast markers. Furthermore, we showed that MAC may affect the ability of TGF-β to induce TβRI phosphorylation. Of note, our data show, for the first time, that MAC, which specifically binds ETRs, also inhibits the ability of TGF-β to induce its direct downstream signaling, resulting in reduced Col1A1 and α-SMA expression.

A possible hypothesis to explain the direct inhibitory effect of MAC on TGF-β signaling is the evidence that TGF-β is able to induce ET-1 production in FBs, and the ability of TGF-β to induce myofibroblast markers is ET-1-dependent [33]. In our experimental setting, MAC, by blocking ET-1 induced after TGF-β stimulation, decreases the expression of both collagen and contractile proteins.

To confirm that the MAC inhibitory effects on TGF-β signaling are mediated by ET-1, we silenced the ET-1 gene in SSc FBs. After silencing, we showed that MAC maintained its inhibiting action on SMAD phosphorylation regardless of ET-1 production, leading us to speculate that the ability of MAC to inhibit TGF-β signaling is independent of ET-1 production. Until some years ago, it was thought that G protein-coupled receptors (GPCRs), like ETRs, and serine/threonine kinase receptors, such as TβRI, along with their respective downstream effectors, represented distinct and linear signaling units that converged on downstream targets. Recently, it has become more clear that GPCR- and serine/threonine kinase receptor-mediated signaling pathways are not mutually exclusive and they may function as partners [36]. The GPCR–receptor tyrosine kinase (RTK) partnerships may result from the activation of RTKs in response to GPCR stimulation [37, 38], thus inducing the formation of GPCR–RTK complexes.

This event has been already described in different biological settings: the platelet-derived growth factor receptor, which is a member of the RTK family, may be found in a tethered complex with the endothelial differentiation gene 1, a member of the GPCR family [39], as well as the insulin receptor (RTK family) and the beta-adrenergic receptors (GPCR family) which may form a functional tethered complex [40, 41].

In our experimental setting, we observed a direct interaction between the ET-1 and the TGF-β receptors, which are members of different receptor families. This interaction includes a physical association, as confirmed by co-immunoprecipitation experiments. Of note, in SSc FBs the co-immunoprecipitated levels were higher than those observed in HC FBs, probably mirroring the evidence that ETARs were significantly higher in SSc FBs. After silencing ETAR, in SSc FB, MAC failed to inhibit the SMAD phosphorylation induced by TGF-β treatment.

We may speculate that this TβRI–ETR complex modulates the effect of ET-1 on the TGF-β specific signaling pathway, leading to the activation of a profibrotic program in FBs. In this scenario, MAC binded in the pocket of the ETR portion of the ET-1/TGF-β receptor complex may induce a conformational change of this complex, responsible for the inhibition of TGF-β signaling. A further speculative and unexplored aspect, in this setting, may be blocking of the TβRI portion of the ET-1/TGF-β receptor complex. However, until now, unlike ET-1, we still do not have any licensed drug able to interfere with TβRI [42], and from a clinical point of view, at present, ET-1 receptor antagonists represent the only possibility we have to modulate this functional complex.

The evidence of this heterogeneous, but functionally active, TβRI–ETR complex suggests the possibility of an antifibrotic effect of MAC, although these preclinical findings need to be translated into the clinical setting. In fact, although many in vitro studies suggested the antifibrotic effect of another ET-1 receptor antagonist, bosentan [29, 32, 33], no clinical benefits were observed when this drug was employed in SSc patients with lung fibrosis [43, 44]. It must be taken into account that the failure of the clinical trials evaluating its efficacy on interstitial lung disease may be biased by the choice of no sensitive primary endpoint, as well as the heterogeneity of the enrolled patients and the lack of histological classifications. On these bases, any further study of ET-1 receptor antagonists in patients with fibrosis might need more stringent inclusion criteria and perhaps use primary and secondary endpoints which might be more responsive to change [45].

## Conclusion

The present study provides the first demonstration, to our knowledge, that some profibrotic effects of ET-1 may reside on a physical coupling of its own receptor with the TβRI, and MAC, binding with this hetero-receptor complex, is able to block the TGF-β-induced signaling, independently of the presence of ET-1.

Our results allow us to speculate that, during SSc, ET-1, accounting for vascular complications, may also contribute to the myofibroblast activation, via the TGF-β machinery, thus linking the early endothelial damage to the subsequent fibrotic process. The MAC inhibitory effect on the TGF-β pathway, here reported, interfering with this activation might suggest new therapeutic perspectives for the treatment of fibrosis, a condition still needing specific therapies.

## Abbreviations

α-SMA: Alpha smooth muscle actin
Col1A1: collagen 1 alpha 1
DMEM: Dulbecco’s modified Eagle’s medium
EC: Endothelial cell
ECM: Extracellular matrix
ET-1: Endothelin-1
ETAR: Endothelin-1 receptor A
ETBR: Endothelin-1 receptor B
ETR: Endothelin receptor
FB: Fibroblast
GPCR: G protein-coupled receptor
HC: Healthy control
MAC: Macitentan
P: Passage
PAH: Pulmonary arterial hypertension
RTK: Receptor tyrosine kinase
scr-siRNA: Scrambled control siRNA
SMAD: Sma and Mad Related
SSc: Systemic sclerosis
TβRI: Transforming growth factor beta type I receptor
TβRII: Transforming growth factor beta type II receptor
TBS/T: Tris-buffered saline/1 % Tween 20
TGF-β: Transforming growth factor beta
UT: Untreated
